# Pharmacological inhibition of NaV1.8 by suzetrigine reveals potent analgesic potential without tolerance development in mice

**DOI:** 10.1186/s13041-025-01253-3

**Published:** 2025-11-13

**Authors:** Md Yousof Ali, Flavia T.T. Antunes, Sun Huang, Lina Chen, Gerald W. Zamponi

**Affiliations:** Department of Clinical Neurosciences, Hotchkiss Brain Institute and Alberta Children’s Hospital Research Institute, Cumming School of Medicine, University of Calgary, Calgary, Canada

**Keywords:** Analgesia, TTX-resistant sodium currents, Neuropathic pain, Thermal hyperalgesia

## Abstract

The Voltage-gated sodium channel NaV1.8 is a critical determinant of nociceptive signaling in primary sensory neurons. Here, we evaluated the analgesic potential of suzetrigine, a potent clinically approved NaV1.8 blocker, using electrophysiological, behavioral, and tolerance paradigms in mice. Whole-cell recordings from dorsal root ganglion neurons revealed that suzetrigine inhibited tetrodotoxin (TTX)-resistant sodium currents in a concentration-dependent manner (IC_50_ = 0.35 ± 0.17 μM), consistent with high-affinity NaV1.8 inhibition. In vivo, intraperitoneal administration of suzetrigine significantly reduced nocifensive behaviors in the formalin test, attenuated CFA-induced thermal hypersensitivity, and reversed mechanical hyperalgesia in the partial sciatic nerve injury-induced neuropathy model. Importantly, repeated dosing did not produce tolerance in a chronic administration paradigm. Although suzetrigine showed limited efficacy in clinical trials for neuropathic pain, its robust analgesic effects in mouse models underscore the challenges of translating preclinical findings to human neuropathic pain, while still supporting the potential of NaV1.8-targeted therapies.

## Background and Results

NaV1.8 is a tetrodotoxin-resistant (TTX) sodium channel that plays a key role in peripheral pain signaling [1, 2]. Its selective expression in nociceptive neurons makes it an attractive target for analgesics aimed at reducing excitability without affecting other neuronal populations [3]. Recently, suzetrigine (initially VX-548, commercially Journavx) was approved by the Federal Drug Administration as a potent and selective NaV1.8 inhibitor for clinical use in acute pain [4]. Clinical trials have shown that it provides analgesia in post-surgical acute pain [5–7], whereas efficacy in neuropathic pain conditions is reportedly limited. Despite its clinical promise, preclinical data in traditional animal pain models have been scarce, and systematic evaluations of its efficacy across diverse pain states remain limited, hindering a clear understanding of how its action translates between preclinical models and patients.

In this study, we sought to bridge this gap by evaluating suzetrigine in multiple mouse pain models, including formalin-induced nociception, CFA-induced thermal hypersensitivity, and partial sciatic nerve injury (PSNI), with additional assessments of tolerance. Electrophysiological recordings were also performed in dorsal root ganglion (DRG) neurons from mice to confirm the compound’s mechanism of action. All experiments were conducted under a University of Calgary Institutional Animal Care and Use Committee-approved protocol.

**Fig. 1:**
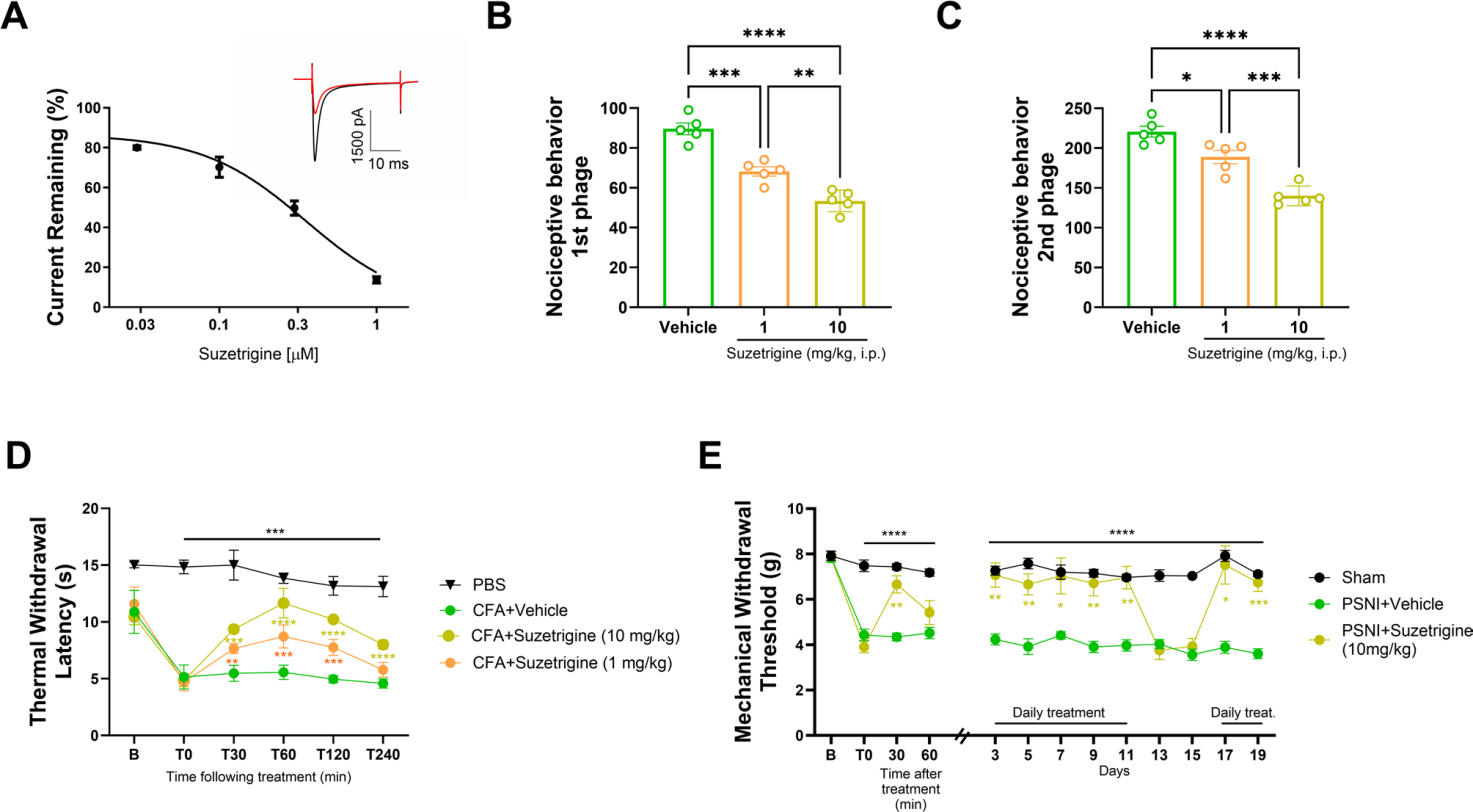
Suzetrigine reduces nociceptive behavior and alleviates inflammatory and neuropathic pain in mice. ** A** Concentration-dependent inhibition of TTX-resistant currents by suzetrigine on native channels in mouse DRG neurons. The neurons were held at − 90 mV and depolarized to the test potential of 10 mV. 500 nM TTX was added to the bath solution consisting of (in mM, pH 7.4) 140 NaCl, 2 CaCl2, 1 MgCl2; 5 KCl, 20 HEPES, and 10 Glucose. The pipette solution contained (in mM, pH = 7.4) 140 CsCl, 10 NaCl, 1 EGTA, 2 Mg-ATP, and 20 HEPES). Data are expressed as a percentage of the current block. The inset shows a representative current trace in the absence and presence (red trace) of 300 nM suzetrigine. **B**–**C** Preventative administration of suzetrigine (1 or 10 mg/kg, i.p., 30 min before formalin injection) in the (**B**) early neurogenic and (**C**) late inflammatory phases of the formalin test. **D** Time-course of suzetrigine analgesic effect in CFA-induced thermal hyperalgesia (Hargreaves test). **E** Long-term effect/tolerance of suzetrigine on the mechanical allodynia assessed by the Dynamic Plantar Aesthesiometer in the partial sciatic nerve injury (PSNI) model. Data are mean ±  SEM. Statistical analysis was performed by one-way or two-way ANOVA, followed by Tukey’s post hoc test. *p <  0.05, **p <  0.01, ***p <  0.001, ****p <  0.0001, compared to vehicle or as indicated

We first tested the ability of suzetrigine to block native mouse NaV1.8 currents from naïve C57BL mice. DRG neurons were isolated as described by us previously [8] and TTX-resistant sodium currents were isolated by blocking TTX-sensitive currents with 500 nM TTX. Sodium currents were elicited by step depolarization and the effect of suzetrigne was examined by application of increasing concentrations. Such an electrophysiological analysis in naïve mouse DRG neurons demonstrated that suzetrigine inhibited TTX-resistant Na^+^ currents in a concentration-dependent manner, with an IC_50_ of 0.35 ± 0.17 µM and approximately 50% inhibition at 300 nM (Fig. 1A). This potent block of TTX-resistant currents, which is expected to be primarily carried by NaV1.8 implies that suzetrigine can effectively suppress a sodium current that is critical for action potential firing in these neurons, thereby reducing nociceptor excitability. It is expected that a similar effect would be observed in neurons isolated from mice with chronic pain, although this was not tested here explicitly in electrophysiological recordings, but instead in in vivo pain models. That said, previous in vitro studies have also shown that suzetrigine selectively inhibits NaV1.8 channels and action potential firing in human DRG sensory neurons [9, 10], with an IC_50_ of 0.68 ± 0.16 nM. Thus, NaV1.8 currents in human DRG displayed markedly higher sensitivity, corresponding to ~ 500-fold greater potency. This could potentially be due differences in the amino acid sequences of human and mouse NaV1.8 channels.

In behavioral assays, suzetrigine demonstrated robust analgesic effects across all models tested. In the formalin test, nocifensive behaviors were elicited by intraplantar injection of 2.5% formalin (20 µL) [11], and then the duration of both the early neurogenic phase and late inflammatory phase of paw licking and biting was measured. Both 1 and 10 mg/kg (i.p., 30 min before intraplantar injection) significantly reduced nociceptive behavior during both phases (*p*<0.001 and *p*<0.0001 in the neurogenic phase, *p*<0.05 and *p*<0.0001 in the inflammatory phase respectively) when compared to vehicle (1% dimethyl sulfoxide in 2-hydroxypropyl-β-cyclodextrin 2.8%) (Fig. 1B, C). This indicates that suzetrigine can effectively prevent pain responses arising from both direct nociceptor activation and subsequent inflammatory processes. In the CFA-induced thermal hypersensitivity model (i.e., intraplantar injection of CFA or PBS vehicle 48 h before behavioral testing) [12], suzetrigine produced a time- and dose-dependent analgesic effect. Administration of 10 mg/kg significantly attenuated thermal hypersensitivity post-injection (*p*<0.0001 compared to vehicle-treated animals), whereas the lower 1 mg/kg dose elicited a less pronounced and shorter-lasting effect (*p*<0.001 compared to the vehicle group) (Fig. 1D). These results suggest that suzetrigine’s efficacy is both dose-dependent and sustained over several hours.

In the PSNI model (loose ligature of the sciatic nerve) [13], a single 10 mg/kg injection at day 14 post-surgery rapidly (within 30 min) and nearly completely increased mechanical withdrawal towards baseline levels (*p*<0.01 when compared to the vehicle group). Interestingly, this effect waned much more quickly compared to what we had observed in the CFA model, such that analgesia was absent at the 60-minute time point (*p*>0.05). Daily administration over 11 days consistently alleviated hypersensitivity without the appearance of analgesic tolerance. Termination of administration of suzetrigine resulted in return to mechanical hypersensitivity that was as pronounced as the original level after surgery. Reinstatement of suzetrigine delivery after this treatment pause between days 12–16 led to a full restoration of the analgesic effects on days 17–19. These findings indicate that repeated suzetrigine treatment does not induce tolerance under the dosing regimen tested. Taken together, the behavioral data across acute, inflammatory, and neuropathic pain models demonstrate a broad spectrum of analgesic activity for suzetrigine in mice.

Our findings provide a mechanistic and behavioral framework supporting suzetrigine’s analgesic potential. Electrophysiological recordings link NaV1.8 inhibition to reduced DRG neuron excitability, while behavioral assays confirm analgesia in diverse pain modalities. Compared to the existing literature, this study fills a critical gap, as preclinical animal data for suzetrigine have been largely absent. Importantly, the absence of tolerance in the PSNI model, together with known safety data, supports the compound’s potential as a non-opioid analgesic.

Earlier this year, suzetrigine was approved by the US Food and Drug Administration (FDA) as the first medication in its class for the treatment of moderate to severe acute pain, based on supporting clinical trials. Nonclinical safety evaluations in rats and monkeys focused on toxicity and addictive potential rather than traditional pain models. The limited availability of preclinical data in animal pain models reflects the compound’s development strategy, likely influenced by its novel mechanism of action and the urgent need for non-addictive analgesics. In a subsequent clinical trial, suzetrigine did not perform better than placebo in a phase 2 trial in lumbosacral radiculopathy (LSR), a neuropathic pain condition [14]. In contrast, our data presented here indicate potent efficacy in a neuropathic pain model. It is difficult to reconcile these disappointing clinical results with the robust reduction in mechanical hypersensitivity observed in our neuropathic pain experiments. Placebo effects in mice and humans differ significantly, and this may contribute to the observed differences between preclinical and human studies. It is also possible that NaV1.8 channels contribute differentially to the development of pain in LSR and sciatic nerve injury-induced neuropathic pain states. Finally, we note that the analgesic effect in our PSNI model was short-lasting, and perhaps such short-lasting relied could have been missed in human studies.

In our study, suzetrigine was effective in mice at 10 mg/kg i.p., corresponding to a human equivalent dose of ~ 0.8 mg/kg (~ 49 mg for a 60 kg adult—for a parenteral equivalent) based on body surface area scaling [15]. In contrast, in clinical trials, adults received 100 mg initially, then 50 mg every 12 h. This highlights the challenges of translating preclinical efficacy, as differences in pharmacokinetics, absorption, and human pain complexity can affect dose-response relationships.

Limitations of this study include the exclusive use of male mice and peripheral DRG recordings, which may not fully capture central mechanisms or sex-dependent differences in pain signaling. Nevertheless, these results provide translationally relevant insight into suzetrigine’s mechanism of action and efficacy, complementing clinical observations and in vitro human DRG studies. In summary, suzetrigine demonstrates potent, dose-dependent analgesic effects in multiple mouse pain models, reduces DRG neuronal NaV1.8 currents, and maintains efficacy with repeated administration.

## Data Availability

All data generated or analysed during this study are included in this published article. Source data will be made available upon reasonable request.

## Abbreviations

CFA: Complete freund’s adjuvant
DRG: Dorsal root ganglion
TTX: Tetrodotoxin
i.p.: Intraperitoneal
LSR: Lumbosacral radiculopathy
PSNI: Partial Sciatic Nerve Injury
